# The MADS-box Transcription Factor PsMAD1 Is Involved in Zoosporogenesis and Pathogenesis of *Phytophthora sojae*

**DOI:** 10.3389/fmicb.2018.02259

**Published:** 2018-09-24

**Authors:** Long Lin, Wenwu Ye, Jiawei Wu, Mingrun Xuan, Yufei Li, Jian Gao, Yonglin Wang, Yan Wang, Suomeng Dong, Yuanchao Wang

**Affiliations:** ^1^Department of Plant Pathology, Nanjing Agricultural University, Nanjing, China; ^2^The Key Laboratory of Integrated Management of Crop Diseases and Pests, Ministry of Education, Nanjing, China

**Keywords:** MADS-box transcription factor, *Phytophthora sojae*, sporangia cleavage, pathogenicity, CRISPR/Cas9, gene knockout

## Abstract

Transcriptional regulation is critical for plant pathogen development and virulence. MADS-box transcription factors belong to a highly conserved transcriptional regulator family in eukaryotic organisms that are involved in various important biological processes. Only one predicted MADS-box gene, *PsMAD1*, was identified in *Phytophthora sojae*, which was highly expressed during the sporangia and infection stages. To investigate its function, we generated *PsMAD1* knockout mutants using the CRISPR/Cas9 system. Compared with the wild-type strain, the mutants showed no changes in vegetative growth, oospore production, or no differences in sensitivity to various abiotic stresses. Although sporangia production was normal, no zoospore release was detected in *PsMAD1* mutants. Microscopy analyses revealed failure of cleavage of the cytoplasm into uninucleate zoospores in the mutants. In addition, the mutants showed reduced virulence in soybean. RNA-seq data indicated that PsMAD1 may regulate many zoospore development and infection associated genes. Thus, *PsMAD1* may be a major regulator of *P. sojae* involved in zoosporogenesis and pathogenesis.

## Introduction

Oomycetes are fungus-like eukaryotic microorganisms belonging to the stramenopile kingdom. They are evolutionarily closely related to photosynthetic algae such as brown algae and diatoms but are distant from fungi. The genetic makeup and metabolism of oomycetes differ from fungi so that many fungicides cannot control oomycete pathogens effectively (Latijnhouwers et al., 2003; Tyler et al., 2006). Among oomycetes, the *Phytophthora* genus contains large amount economically significant pathogens in agriculture. For example, soybean (*Glycine max* L.) root and stem rot caused by *Phytophthora sojae* is one of the most destructive diseases in soybean production, with an annual cost of $1–2 billion worldwide (Tyler, 2007).

The main vectors of *P. sojae* dispersal are oospores and zoospores. Oospores have a thick-walled survival structure for sexual reproduction, which is responsible for their survival in extreme environments and ability to trigger new epidemics in subsequent growing seasons (Judelson and Blanco, 2005). Zoospores are an asexual reproduction structure and the most important route of infection of roots, especially in flooded soil. Zoosporogenesis involves the formation of sporangia, which may be triggered by environmental changes such as flooding, after which the sporangial cytoplasm is cleaved and the nuclei are separated, resulting in the formation of zoospores (Judelson and Blanco, 2005). Biflagellate wall-less zoospores are released and swim chemotactically toward isoflavones released by soybean roots to start infection (Morris and Ward, 1992). When zoospores encounter the plant surface, they transform into adhesive cysts and then germinate to produce hypha to penetrate plant. Infection hypha is able to grow in the intercellular space of the host cells in a biotrophic phase during early infection. At this stage, many secreted proteins (including effectors) are delivered into apoplast and cytoplasm of plant cells through haustoria, which can degrad physical barrier and suppress host immunity during infection (Blackman et al., 2014). After 12 h, the pathogen enters a necrotrophic growth phase, invading directly into host cells, spreading quickly throughout host tissues, causing large, water-soaked, and necrotic lesions (Moy et al., 2004). Then oospores are produced abundantly in infected tissues and they are able to survive for years in the soil until epidemic period (Judelson and Blanco, 2005).

The ability of organisms to grow, differentiate, and respond to environmental cues relies largely on the regulation of gene expression. Transcription factors (TFs) are a large family of *trans*-acting molecules that have key roles in gene expression regulation. MADS-box genes encode a conserved family of TFs in nearly all eukaryotes, which are important in diverse biological functions, especially development (Messenguy and Dubois, 2003). MADS-box genes are named after the five founding members of the family: Mcm1 (yeast) (Passmore et al., 1989), Arg80 (yeast) (Dubois et al., 1987) or agamous (plant) (Yanofsky et al., 1990), deficiens (plant) (Sommer et al., 1990), and serum response factor (SRF, human) (Norman et al., 1988). The MADS-box TF contains a 55–60-amino acid (aa) conserved DNA binding and dimerization domain named the MADS-box domain, usually found in the N-terminal region (Schwarz-Sommer et al., 1990). Based on the domain sequence and phylogeny, MADS-box family members can be divided into two types: SRF type and myocyte enhancer factor 2 (MEF2) type (Alvarez-Buylla et al., 2000). Both recognize AT-rich consensus sequences, but the SRF-type family binds as a homo-dimer to a 10-bp consensus sequence CC(A/T)_6_GG (CArG-box or SRF site) (Treisman, 1990), whereas the MEF2-type family binds as a homo- or hetero-dimer to a 10-bp consensus sequence CTA(A/T)_4_TAG (MEF2 site) (Pollock and Treisman, 1991).

The functions of some MADS-box TFs have been reported in fungal plant pathogens. For example, *Magnaporthe oryzae* MEF2-type TF MIG1 is required for differentiation of secondary infectious hyphae in host cells (Mehrabi et al., 2008), and SRF-type TF MCM1 is required for virulence as well as sexual and asexual reproduction (Zhou et al., 2011). In *Fusarium verticillioides*, both SRF-type TF *MADS1* and MEF2-type TF *MADS2* are required for sexual reproduction, but not virulence, and a *MADS1* mutant showed reduced vegetative growth and fumonisin B1 production (Ortiz and Shim, 2013).

To our knowledge, the functions of MADS-box TFs have not been reported in oomycetes. In this study, we predicted only one MADS-box gene, *PsMAD1*, in *P. sojae*. According to digital gene expression profiling data (Ye et al., 2011), *PsMAD1* showed highly induced expression levels during the sporangia and infection stages. We knocked out *PsMAD1* using CRISPR/Cas9 system and detected defects in development and virulence phenotypes in the mutants.

## Results

### Expression Pattern and Phylogenetic Analysis of *PsMAD1*

**FIGURE 1:**
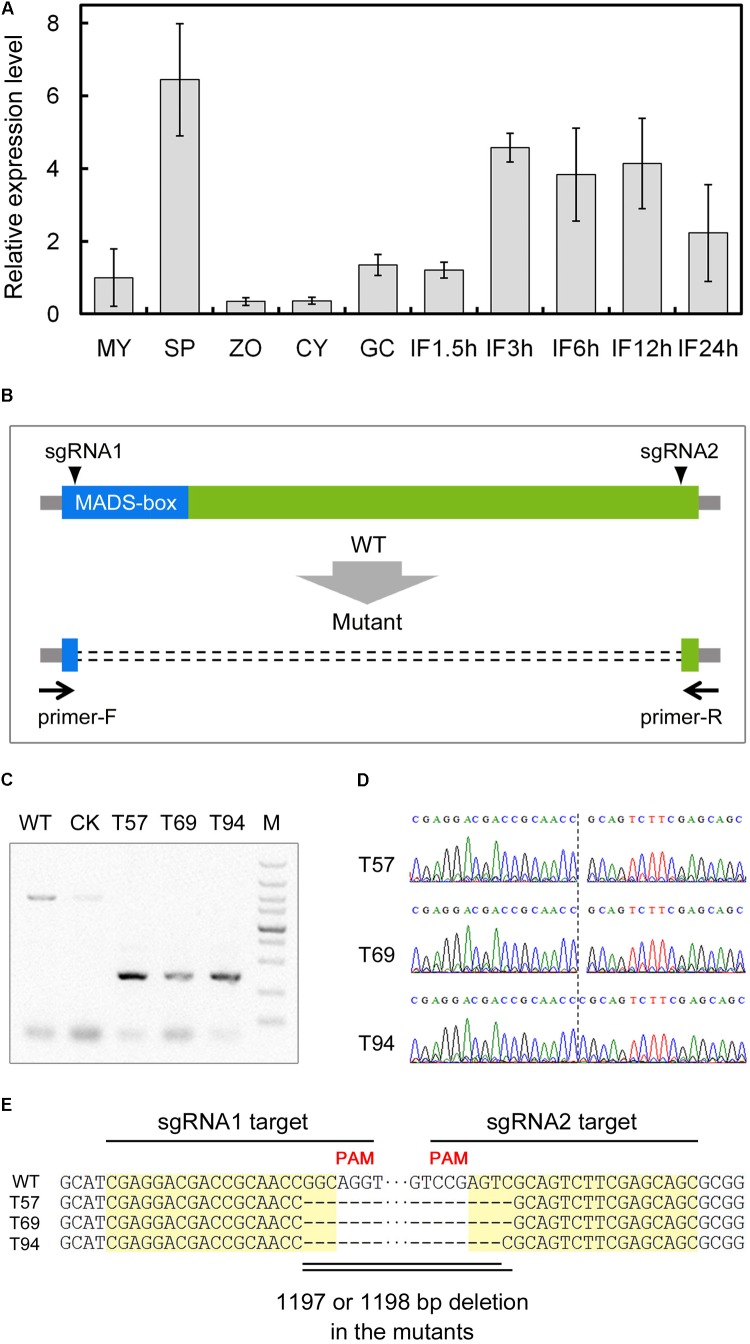
Expression profiling and gene knockout of *PsMAD1*. **(A)** Expression pattern of *PsMAD1* during the asexual life cycle and infection stages. Expression levels were determined by quantitative real-time PCR using RNAs extracted from vegetative mycelia (MY), sporangia (SP), zoospores (ZO), cysts (CY), germinated cysts (GC), and during infection stages (IF 1.5, 3, 6, 12, and 24 h). Relative expression levels were calculated using the MY values as a reference. **(B–E)** CRISPR/Cas9-mediated mutagenesis of *PsMAD1*. Two single guide RNAs targeting the two sides of the *PsMAD1* coding region were designed to disrupt *PsMAD1* **(B)**. Three independent truncated mutants were identified by genomic PCR **(C)** and confirmed by sequencing **(D,E)**.

To characterize the regulatory network of *P. sojae* development and infection, we analyzed the expression patterns of predicted TFs based on global digital gene expression profiling data (Ye et al., 2011). We identified a MADS-box TF candidate gene (*Ps143579*, named *PsMAD1*) that was highly expressed during the sporangia and infection stages, and confirmed the expression pattern with quantitative reverse transcription (qRT)-PCR (**Figure 1A**). This indicates that *PsMAD1* may have roles during sporangia development and infection.

According to the sequences cloned from mycelia genomic (g)DNA and complementary (c)DNA, we determined that the *PsMAD1* gene was 1604 bp long with three introns (87, 80, and 90 bp in length; GenBank: ACG80381.1). *PsMAD1* encoded a 448-aa protein with a predicted MEF2-type MADS-box domain (NCBI Conserved Domain Database accession ID: cd00265; aa 1–60) in the N-terminus. Based on BLAST searches with representative MADS-box TF proteins from *Saccharomyces cerevisiae* (MCM1, SRF-type; RLM1, MEF2-type), we identified only one homolog in each genome of analyzed oomycete species. A phylogenetic tree constructed using full-length protein sequences of PsMAD1 and the homologs from oomycetes and fungi showed that the oomycete MADS-box TF proteins were conserved and the clade of them were close to that of RLM1, thus they likely belong to the MEF2-type family (**Supplementary Figure S1**).

### CRISPR/Cas9 Genome Editing for *PsMAD1* Knockout

To investigate the functions of *PsMAD1*, we generated knockout mutants of *P. sojae* strain P6497 using the CRISPR/Cas9 system. Two single guide (sg)RNAs were designed to disrupt the *PsMAD1* coding region (**Figure 1B**) using a previously described protocol (Fang and Tyler, 2016). Two sgRNA vectors and the *hSPCas9* vector (expressing CAS9) were co-transformed into *P. sojae* using polyethylene glycol (PEG)-mediated protoplast transformation (Hua et al., 2008). After gDNA PCR and sequencing screening, three independent mutants were identified (**Figures 1C,D**). An 1198-bp fragment was removed between the two sgRNA target sites in the mutants T57 and T69, whereas an 1197-bp fragment was removed from T94 (**Figure 1E**). A transformant that failed to acquire *PsMAD1* mutation was selected as the control (CK) strain (**Figure 1C**).

### *PsMAD1* Deletion Affects Zoosporogenesis in *P. sojae*

Compared to the wild type (WT) and CK, the mutants showed no significant differences of growth rate on V8 medium or V8 medium supplemented with 2.5, 5, or 10 mM H_2_O_2_; 1 or 1.5 mM sorbitol; or 0.3 or 0.6 M NaCl, indicating that the mutants had no mycelial growth defects and no differences in sensitivity to various stresses (**Supplementary Figure S2**). We observed oospore production of the mutants after 10 days of growth on lima bean agar (LBA) medium. The mutants showed similar oospore production as the WT and CK (**Supplementary Figure S3**). Thus, *PsMAD1* is likely not essential for sexual reproduction.

**FIGURE 2:**
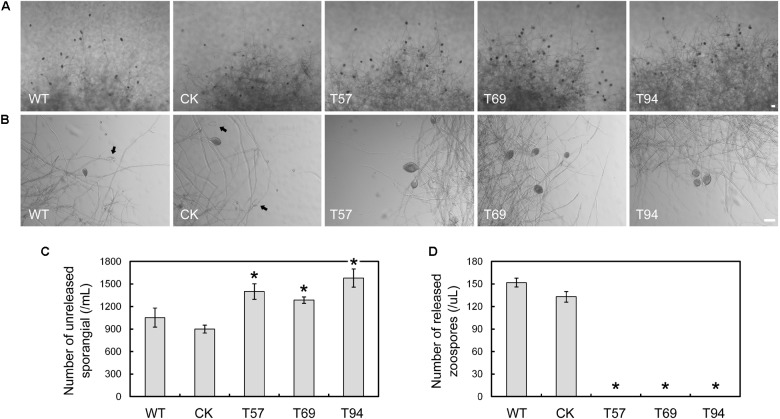
*PsMAD1* is required for zoospore production. Mycelia were cultured in V8 broth medium for 3 days and flushed with water three times. Sporangia and zoospore production were detected after 9 h. The *PsMAD1* mutants showed greater sporangia production than the wild type **(A–C)**, although no zoospores were detected in the mutants **(A,B,D)**. Arrows indicated released sporangia. Scale bar = 50 μm. Asterisks indicate significant differences (*P* < 0.01).

To test zoospore production, the mycelia of mutants, WT, and CK were cultured in liquid V8 medium for 4 days, and then used to induce sporangium formation. Zoospore production was observed 9 h after flushing the mycelia with water. We found that the mycelia of the mutants kept more unreleased sporangia than the WT and CK (**Figures 2A–C**); however, no zoospore production was detected in the mutants (**Figures 2A,B,D**), even after 24 h (data not shown). The results indicate that *PsMAD1* is essential for zoospore development, but not sporangium formation. The greater number of unreleased sporangia in the mutants may have been caused by defects in zoospore release.

### *PsMAD1* Is Requires for Sporangia Cleavage During Zoospore Development

**FIGURE 3:**
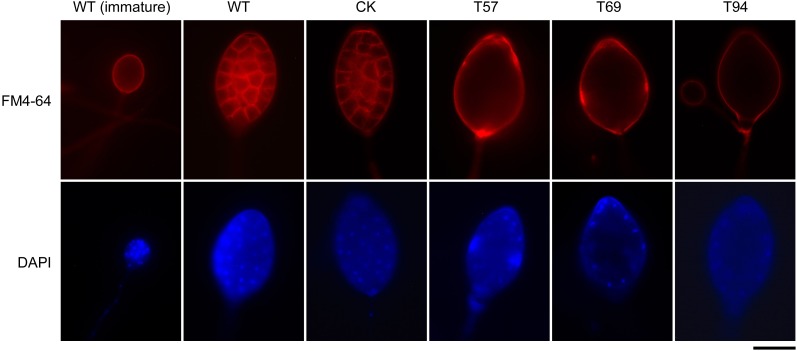
Nuclei distribution and cytoplasm cleavage within sporangia are hindered in *PsMAD1* mutants. The nuclei in sporangia were detected with DAPI staining. Plasma membrane was observed by FM4-64 staining. Scale bar = 20 μm.

Zoospore release involves cleavage of the sporangial cytoplasm by membrane networks and the assembly of two flagella per zoospore (Judelson and Blanco, 2005). Based on fluorescent dye staining and morphology observation of sporangia, we found that there were no significant differences in the cellular structure of sporangia in the early stage; however, although the sporangia of *PsMAD1* mutants were multinucleated with undifferentiated cytoplasm and were of similar size and structure as the WT and CK, sporangial cleavage was impaired in the mutants (**Figure 3**). The nuclei within the sporangia of the WT and CK were regularly spaced, and the cytoplasm differentiated to form developed zoospores. By contrast, the sporangial cytoplasm of the mutants remained undifferentiated and the nuclei remained disordered (**Figure 3**). These results demonstrate that *PsMAD1* is required for sporangia cleavage during zoospore development, leading to the defects in zoospore release.

### *PsMAD1* Is Also Required for Full Virulence of *P. sojae*

**FIGURE 4:**
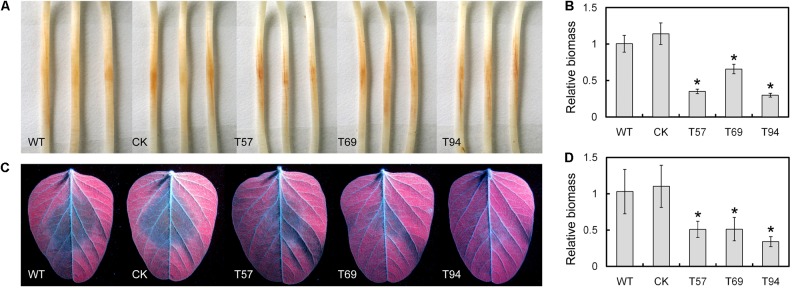
*PsMAD1* mutation results in attenuated virulence. 5 × 5-mm hyphal plugs were inoculated on panel **(A)** hypocotyls or **(C)** leaves of the Williams soybean cultivar and photographed after 2 days. The relative biomass of *Phytophthora sojae* in panel **(B)** hypocotyl infection or **(D)** leaf infection was detected by quantitative real-time PCR. Asterisks indicate significant differences (*P* < 0.01).

Etiolated seedlings of the Williams soybean cultivar were inoculated with 5 × 5-mm hyphal plugs of the WT, CK, and mutants, respectively. After 2 days, we found the lesion sizes caused by the mutants were smaller than those of the WT and CK (**Figure 4A**). Biomass quantification confirmed that the relative amount of *P. sojae* DNA in the inoculated soybean hypocotyls was significantly reduced in the mutants relative to the WT and CK (**Figure 4B**). In addition, we tested the virulence of these strains to leaves, and obtained similar results (**Figures 4C,D**). These results suggest that *PsMAD1* is required for full virulence of *P. sojae* in soybean infection.

### PsMAD1 May Regulate Many Zoospore Development and Infection Associated Genes

To identify *P. sojae* genes regulated directly or indirectly by PsMAD1, we performed RNA-seq to compare the transcriptomes of the WT and the mutants T57 and T94. Samples were collected at the sporangia stage (6 h after flushing the mycelia with water; SP) and infection stage (samples were collected at 1.5, 3, 6, 9, and 12 h post-infection on etiolated soybean seedlings, and equal volume of infected hypocotyls were mixed; IF). Two independent biological replicates were analyzed. There were few differentially expressed genes (DEGs) between each biological replicate pairs and between the two mutants, indicating that the data for the replicates were robust. In both mutant libraries, *PsMAD1* transcripts were truncated, confirming that *PsMAD1* was knocked out in T57 and T94 (**Supplementary Figure S4A**).

We identified 280 and 44 DEGs in the mutants compared to the WT at sporangia stage (SP-DEGs) and infection stage (IF-DEGs), respectively. Because the infection stage libraries were obtained from mixed pathogen and plant tissues, only 4–6% of the transcript reads were from *P. sojae*, significantly less than those in the sporangia stage libraries (>78%; **Supplementary Table S1**), and less DEGs were identified for the infection stage. qRT-PCR results of some selected DEGs further confirmed reliability of the RNA-seq data (**Supplementary Figures S4B–E**).

**FIGURE 5:**
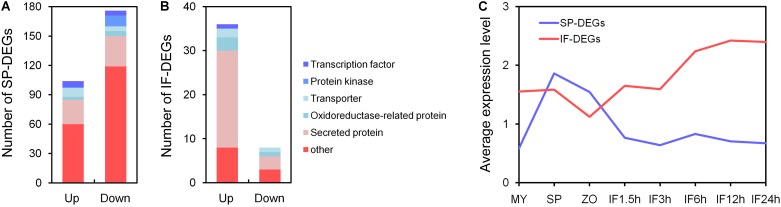
Function annotation of differentially expressed genes. **(A,B)** Function annotation of DEGs in SP and IF samples. **(C)** Average transcript levels of the DEGs in SP and IF samples during asexual development and the infection stages: vegetative mycelia (MY), sporangia (SP), zoospores (ZO), and during infection stages (IF 1.5, 3, 6, 12, and 24 h).

Among the 280 SP-DEGs, 104 genes (37%) were up-regulated and 176 genes (63%) were down-regulated in the mutants compared to the WT; 36% of the SP-DEGs were predicted to encode secreted proteins, oxidoreductase-related proteins, transporters, protein kinases, and TFs, which may involved in signal transduction during sporangia development; however, the majority of the other genes were factional unknown (**Figure 5A**). Among the 44 IF-DEGs, 36 genes were up-regulated, and 8 genes were down-regulated in the mutants compared to the WT (**Figure 5B**). There were 22 up-regulated genes and 3 down-regulated genes had predicted signal peptides, including those predicted to encode RxLR effectors, proteases, and carbohydrate-active enzymes (CAZymes). These genes may be involved in the interaction between *P. sojae* and soybean. Detail information of DEGs is listed in **Supplementary Table S2**.

Based on the gene expression profile data for the detailed developmental and infection stages of wild type *P. sojae* (Ye et al., 2011), we found that the expression of SP-DEGs were specifically induced during the sporangia, zoospore stages in WT, and that of IF-DEGs were relatively higher during the germinating cyst and infection stages in WT (**Figure 5C**). The results indicated that the phenotypic defect of *PsMAD1* knockout mutants in zoosporogenesis and pathogenesis may due to the abnormal expression of SP-DEGs and IF-DEGs, respectively.

## Discussion

MADS-box proteins are widespread in fungi pathogens, involving in development, metabolism and virulence (Zhou et al., 2011; Ortiz and Shim, 2013). Only one conserved MADS-box protein, which belongs to MEF2 type, can be identified from each oomycete genome. We knocked out *PsMAD1* and found it was essential for asexual development and virulence, not vegetative growth and sexual development in *P. sojae*. This result was in accordance with the expression pattern of *PsMAD1*, suggesting that *PsMAD1* was a key regulator of zoosporogenesis and pathogenesis.

Zoospore release, which is important for the *Phytophthora* pathogenic life cycle, involves cleavage of the sporangial cytoplasm by membrane networks and the assembly of two flagella per zoospore (Judelson and Blanco, 2005). Our data showed that *PsMAD1* mutation led to defects in sporangia cleavage, and 280 SP-DEGs were identified. Among these genes, the protein phosphatase coding gene, *PsCDC14* (*Ps108222*), showed down-regulation. *PsCDC14* and its homolog *PiCDC14* have been revealed to be essential for zoosporogenesis in *P. sojae* and *Phytophthora infestans*, respectively (Ah Fong and Judelson, 2003; Zhao et al., 2011). *PiCDC14* acts as a transcription regulator to reprogram gene expression during zoospore formation (Seidl et al., 2013). In *Schizosaccharomyces pombe*, the CDC14p-like protein, CLP1p, controls cell cycle-specific gene expression by binding to and dephosphorylating the MADS-box protein MBX1p (Papadopoulou et al., 2010). *PsMAD1* may interact with and regulate the transcription of *PsCDC14* to regulate the transcriptome in sporangia.

In *Dictyostelium discoideum*, cAMP-dependent protein kinase (PKA) is upstream of MADS-box TF *SRFA* in the sporulation-associated signaling pathway. Activation of PKA leads to the induction of *SRFA* expression, which is essential for the normal expression of several spore-specific genes (Escalante and Sastre, 2002). We found three SP-DEGs encoding PKA and one SP-DEG encoding regulatory subunit of PKA, suggesting that they may serve as feedback of PsMAD1 regulation in the cAMP signaling pathway of *P. sojae*. Calcium signaling is essential for zoosporogenesis in oomycete. In *P. infestans*, a recent RNA-seq analysis revealed that the transcription of two-thirds of genes induced during zoosporogenesis relied on calcium signaling (Ah-Fong et al., 2017). Inhibitors of calcium pathways strongly inhibited zoospore release in *P. infestans* (Judelson and Roberts, 2002). We found six SP-DEGs encoding calcium-binding proteins (including three SP-DEGs encoding kinases) that were down-regulated, indicating that PsMAD1 may also have a role in the calcium signaling pathway.

*Phytophthora sojae* encodes hundreds of secreted proteins (effectors) to attack soybean immunity (Wang and Wang, 2018). Secreted proteins identified in *P. infestans* mainly involve in cell wall modifications, pathogenesis, defense responses, and proteolytic processes (Meijer et al., 2014). Among the 40 identified PsMAD1-regulated IF-DEGs, several genes encoding CAZymes and proteases likely involved in plant cell wall degradation even plant immunity suppression were down-regulated. For example, the Carbohydrate Esterase 1 (CE1) family member Ps109641 is a feruloyl esterase, which may involve in degrading host pectin and xylan (de Vries et al., 2002). Ps143289 is a protease; in *Pseudomonas syringae*, effector AvrRpt2 which encodes a cysteine protease can cleave RIN4 from *Arabidopsis* membranes and block RPM1 activation to suppress plant immunity (Kim et al., 2005). Reactive oxygen species (ROS) produced by host plant is a kind of early defense response to suppress pathogen, and plant pathogens also possess various antioxidant enzymes to counter the threat of oxidative damage (Chauhan et al., 2006). Ps109679 is a NADH-dependent reductase, which may involve in quench ROS derived from hosts. In addition, many secretome coding genes were transcriptionally up-regulated, including three RxLR effector genes (**Figure 5C**). The RxLR effector PsAvh238 in P6497 can both trigger and suppress plant immunity, which is essential for full virulence (Yang et al., 2017). *PsMAD1* may directly or indirectly regulate the accurate expression of these effectors for successful *P. sojae* infection.

As a TF, MADS-box proteins can bind to specific motifs in promoter regions to regulate gene expression, where SRF types bind to a 10-bp consensus sequence CC(A/T)_6_GG (Treisman, 1990) and MEF2 types bind to a 10-bp consensus sequence CTA(A/T)_4_TAG (Pollock and Treisman, 1991). To predict binding site(s) of PsMAD1, we used MEME software to discover conserved motifs in the promoter regions of the DEGs (**Supplementary Figure S5**). However, both classic MADS-box binding sites were not identified. Comparing with the enriched motifs (in the promoter sequences of the SP-DEGs or IF-DEGs) with reported stage-specific promoter motifs in *P. infestans* (Roy et al., 2013), we found motif SP-1 was similar to mycelia and sporangia stage-specific motif CTTCAAC, and motifs IF-2 and IF-3 were similar to infection stage-specific motif TACATGTA and AGC[AG]CAAG, respectively. These results suggested that the promoter motifs bound by PsMAD1 may differ in *P. sojae*. In addition, transcription of several TFs was influenced by *PsMAD1* mutation; therefore, PsMAD1 may also regulate the transcriptome by interacting with or regulating these TFs.

## Conclusion

In summary, we identified a conserved oomycete MADS-box protein and studied the function of PsMAD1 in *P. sojae* with a CRISPR/Cas9-mediated knockout. *PsMAD1* mutation led to defects in zoosporogenesis, reductions in virulence, and differential expression of many zoospore development and infection associated genes. This study provides new data to improve our understanding of transcriptional regulation in oomycetes. Further work should focus on identifying the interaction between the proteins and binding motifs of *PsMAD1* to explain the mechanism of *PsMAD1* transcriptional regulation. MADS-box proteins may be conserved and also important for zoosporogenesis and pathogenesis in other oomycetes, making them a potential fungicide target for oomycete pathogen control.

## Experimental Procedures

### Phylogenetic Analysis of MADS-box Proteins

All oomycete and fungal MADS-box protein sequences were obtained from NCBI^1^. The obtained oomycete sequences were submitted to NCBI-CDD^2^ and SMART^3^ to identify conserved functional domains. Sequence alignments were created with the ClustalW program (Thompson et al., 1994) and the phylogenetic tree was constructed with the MEGA 6.0 (Tamura et al., 2013) program with a neighbor-joining algorithm using 1,000 bootstrap replicates.

### *Phytophthora sojae* Strains and Culture Conditions

*Phytophthora sojae* strain P6497 and CRISPR/Cas9-related vectors were provided by Professor Brett Tyler (Oregon State University, United States). All strains in this study were routinely grown on V8 medium at 25°C in the dark. Vegetative mycelia (MY), sporangia (SP), zoospores (ZO), cysts (CY) and germinated cysts (GC), as well as infection stages (IF 1.5, 3, 6, 12, and 24 h), were collected as described previously (Ye et al., 2011).

For the growth rate analysis, 5 × 5-mm hyphal plugs were inoculated on V8 medium plates and the diameter of each line was measured after 7 days. Sensitivity to different stresses was evaluated on plates with V8 medium or V8 medium supplemented with 2.5, 5, or 10 mM H_2_O_2_; 1 or 1.5 M sorbitol; or 0.3 or 0.6 M NaCl. Fresh 5 × 5-mm hyphal plugs were inoculated on V8 medium plates and the diameter of each line was measured after 7 days, and the inhibition rate = (growth rate on plates without stress–growth rate on plates with stress)/growth rate on plates without stress. The experiments were repeated three times for each assay in triplicate. The data were subjected to statistical analysis based on a two-tailed *t*-test (*P* < 0.01).

### Nucleic Acid Manipulation and Quantitative PCR Assay

Genomic DNA of the *P. sojae* strains was isolated using a plant DNA kit (TIANGEN) from mycelia grown in V8 liquid medium or infected soybean for the gDNA PCR or biomass assay, respectively. RNA was extracted using the EZNA total RNA kit I (Omega). cDNA was synthesized with PrimeScript First Strand cDNA Synthesis Kit (TaKaRa Bio Inc.) following the manufacturer’s protocol. Quantitative PCR was performed in 20-μL reactions containing 20 ng of DNA, 0.2 mM of primers for the target gene or reference gene, 10 μL of SYBR Premix ExTaq (TaKaRa Bio Inc.), and 6.8 μL of ddH_2_O. PCR was performed on an ABI Prism 7500 Fast Real-Time PCR System (Applied Biosystems Inc.) under the following conditions: 95°C for 30 s, followed by 40 cycles of 95°C for 5 s, and 60°C for 34 s, and finally 95°C for 15 s, 60°C for 1 min, and 95°C for 15 s. Relative expression levels were calculated using the 2^−[Ct(targetgene) − Ct(referencegene)]^ method. For the gene expression assay, the actin gene (*PsActA* = *Ps108986*) from *P. sojae* was used as a constitutively expressed endogenous control. To quantify the *P. sojae* biomass in infected soybean tissue, *P. sojae PsACTA* and soybean *GmCYP2* (NC_016099.2) were quantified with qPCR, and the biomass ratio was calculated using the 2^−[Ct(*PsACTA*) − Ct(Gm*CYP*2)]^ method. All experiments were performed at least three times with independent RNA isolations. The data were subjected to statistical analysis based on a two-tailed *t*-test (*P* < 0.01).

### CRISPR/Cas9 Editing for *PsMAD1* Knockout

**Supplementary Table S3** lists the primers used in this study. Two sgRNAs (sgRNA1 and sgRNA2) were selected using a standard as described previously (Fang and Tyler, 2016). The targets of sgRNA1 and sgRNA2 were shown in **Figure 1E**. Potential off-target sites were examined using the FungiDB^4^ alignment search tool (BLASTN) against the *P. sojae* genome. sgRNA1 and sgRNA2 were cloned into sgRNA vector pYF2.3G-Ribo-sgRNA, respectively. Two sgRNA vectors and the *hSPCas9* vector pYF2-PsNLS-hSpCas9 were co-transformed into *P. sojae* using PEG-mediated protoplast transformation (Hua et al., 2008). After G418 resistance and green fluorescence screening, gDNA was extracted from the transformants. *PsMAD1*-specific primers (*PsMAD1*-Genome-F and *PsMAD1*-Genome-R) were used to amplify the *PsMAD1* gene from the transformant gDNA, and truncated fragments were cloned into T-simple plasmid (TaKaRa Bio Inc.) for sequencing.

### Analysis of Zoospore and Oospore Development

For zoospore production, five 5 × 5-mm hyphal plugs were inoculated on 8 mL of V8 broth. Sporangia or zoospores were prepared by repeatedly washing 3-day-old mycelia with sterile water and incubating in 5 mL of water at 25°C until most mycelia developed sporangia, which then released zoospores. Next, 5-μL suspensions were counted for zoospore production after 9 h. The mycelia were homogenized with 5 mL of distilled water, and 5-μL suspensions were counted for sporangia production. For oospore production, 5 × 5-mm hyphal plugs were induced on LBA medium plates at 25°C for 10 days. Then, 2 × 2-cm hyphal plugs near the incubation points were homogenized with 5 ml of water, and 5-μL suspensions were counted for sporangia production. All experiments were performed at least three times independently. The data were subjected to statistical analysis based on a two-tailed *t-*test (*P* < 0.01).

To visualize the distribution of nuclei, sporangia were stained with the blue-fluorescent nucleic acid stain 4′,6-diamidino-2-phenylindole (DAPI), dilactate (Invitrogen), and then viewed using an Olympus 1 × 71 inverted microscope at 330–385 nm. Red-fluorescent FM 4-64 dye (Invitrogen), which selectively binds to the plasma membrane, was used to monitor the cleavage system in sporangia.

### Virulence Assay

Virulence was tested after hyphal plug inoculation of the hypocotyls of etiolated soybean seedlings or leaves of the Williams cultivar, a cultivar compatible with *P. sojae* strain P6497. Soybeans grown in a greenhouse at 25°C with a 16-h/8-h light/dark cycle for 14 days and leaves from the second-leaf stage were used for leaf infection and soybeans grown in the dark at 25°C for 4 days were used for hypocotyl infection. Then, 5 × 5-mm hyphal plugs were inoculated on hypocotyls or leaves and incubated at 25°C in the dark for 2 days before sampling. Pictures of leaves were taken under UV light. Virulence was also quantified by determining the ratio of *P. sojae* DNA to soybean DNA in the infected plants, as measured by qRT-PCR. All assays were repeated independently at least three times. The data were subjected to statistical analysis based on a two-tailed *t*-test (*P* < 0.01).

### RNA-seq Sampling and Sequencing

For the sporangia sampling, WT, T57, and T94 strains were inoculated in V8 broth medium for 3 days and flushed with water three times, and samples were collected 6 h after water flushing. For the infection stages, 5 × 5-mm hyphal plugs were inoculated on the hypocotyls of etiolated soybean seedlings (Williams cultivar). Samples were collected at 1.5, 3, 6, 9, and 12 h post-infection. Five centimeters lengths of infected hypocotyls were collected at the indicated time points, and then mixed hypocotyls as infection sample. Two biological replicates were included per treatment. The samples were frozen immediately in liquid nitrogen and stored at −80°C until RNA extraction. Sequencing libraries were generated using the VAHTS mRNA-seq v2 Library Prep Kit for Illumina (Vazyme) following manufacturer’s recommendations. RNA-seq was conducted using an Illumina Hiseq X Ten platform and 150-bp paired-end module. The names of the libraries were as follows: 57SP1 & 57SP2, 94SP1 & 94SP2, and SP1 & SP2 were the sporangia replicates for T57, T94, and WT, respectively, and 57IF1 & 57IF2, 94IF1 & 94IF2, and IF1 & IF2 were the infection replicates for T57, T94, and the WT, respectively. The RNA-seq data have been deposited in NCBI database (BioProject accession: PRJNA473896).

### RNA-seq Read Mapping and Gene Expression Analysis

Raw reads were filtered by removing reads containing adapter, poly-N, and low-quality reads for subsequent analysis. The clean reads were mapped to *P. sojae* (v1.1 for isolate P6497 ^5^) with TopHat v2.1.1 software^6^. A total of two mismatches and gaps per read were allowed, and the data were only included in analyses when both of a pair of reads were successfully mapped. **Supplementary Table S1** presents the RNA-seq mapping results.

Transcript abundance was indicated as fragments per kilobase of exon model per Million mapped reads. To identify DEGs, the read counts for each gene model were obtained using featureCounts software^7^, and the log_2_ fold change (log_2_FC) value and adjusted *P*-value were calculated using DESeq2 software^8^. Because the reads mapped to *P. sojae* were much lower in infection libraries than in sporangia libraries, for infection libraries, genes with an adjusted *P*-value <0.05 and an absolute log_2_FC ≥ 1 were considered to be differentially expressed, while for sporangia libraries, genes with an adjusted *P*-value <0.001 and an absolute log_2_FC ≥ 2 were considered to be differentially expressed. We also performed differential expression analysis between duplicate samples for all *P. sojae* genes using edgeR software (see text footnote 8) to demonstrate the reproducibility of the experiments. The predicted functions of genes were annotated by querying the non-redundant protein sequences database of NCBI (see text footnote 1) and the PFAM database^9^. The transcript reads of *PsMAD1* were visualized using IGV software^10^. Promoter motifs (in 1000 bp up-stream) of the DEG coding regions were analyzed using MEME software^11^.

## Author Contributions

YuW, SD, WY, and YaW conceived the study. LL, JW, YL, JG, and YoW did the experiments. WY and MX did the phylogenetic and RNA-seq analyses. LL and WY wrote the paper.

## Conflict of Interest Statement

The authors declare that the research was conducted in the absence of any commercial or financial relationships that could be construed as a potential conflict of interest.

## Footnotes

1 http://www.ncbi.nlm.nih.gov

2 http://www.ncbi.nlm.nih.gov/Structure/cdd

3 http://smart.embl-heidelberg.de/

4 http://fungidb.org/fungidb/

5 jgi.doe.gov

6 ccb.jhu.edu

7 bioinf.wehi.edu.au

8 www.bioconductor.org

9 pfam.xfam.org

10 software.broadinstitute.org/software/igv

11 http://meme-suite.org/tools/meme

## Supplementary Material

FIGURE S1 Phylogenetic analysis of putative MADS-box TF proteins. The neighbor-joining tree was constructed based on the amino acid sequences. Bootstrap values greater than 80 were displayed.

FIGURE S2 *PsMAD1* mutants showed similar growth rate and sensitivity to H_2_O_2_, sorbitol, and NaCl as the wild type (WT). Mycelial growth of the WT, control (CK), and mutants on V8 medium plates without or supplemented with different concentration of H_2_O_2_, sorbitol, or NaCl.

FIGURE S3 *PsMAD1* mutants showed similar oospore production as the wild type (WT). **(A)** Oospore production of the WT, control (CK), and mutants. **(B)** Number of oospores per centimeter-squared zone around the inoculation site. All strains were grown for 10 days on lima bean agar medium to induce oospore formation. Scale bar = 200 μm.

FIGURE S4 Transcriptional analysis and verification of indicated genes. **(A)** Transcript reads of *PsMAD1* were truncated in mutant libraries. **(B–E)** Expression levels of selected genes were confirmed by qRT-PCR. Asterisks indicate significant differences (*P* < 0.01).

FIGURE S5 Identified conserved motifs in promoter sequences of PsMAD1-regulated genes. One kilobase promoter sequences of SP-DEGs **(A)** and IF-DEGs **(B)** were used to identify conserved motifs using MEME software, respectively. The top three motifs with most statistical significance were shown.

TABLE S1 Mapping results of the RNA sequencing reads of the indicated libraries.

TABLE S2 Detailed information of differentially expressed genes.

TABLE S3 The primers used in this study.
