# Supplementary figures and images for: The MADS-box Transcription Factor PsMAD1 Is Involved in Zoosporogenesis and Pathogenesis of Phytophthora sojae
Source: Front Microbiol. 2018 Sep 24;9:2259. doi: 10.3389/fmicb.2018.02259 (PMC6165875; doi:10.3389/fmicb.2018.02259)

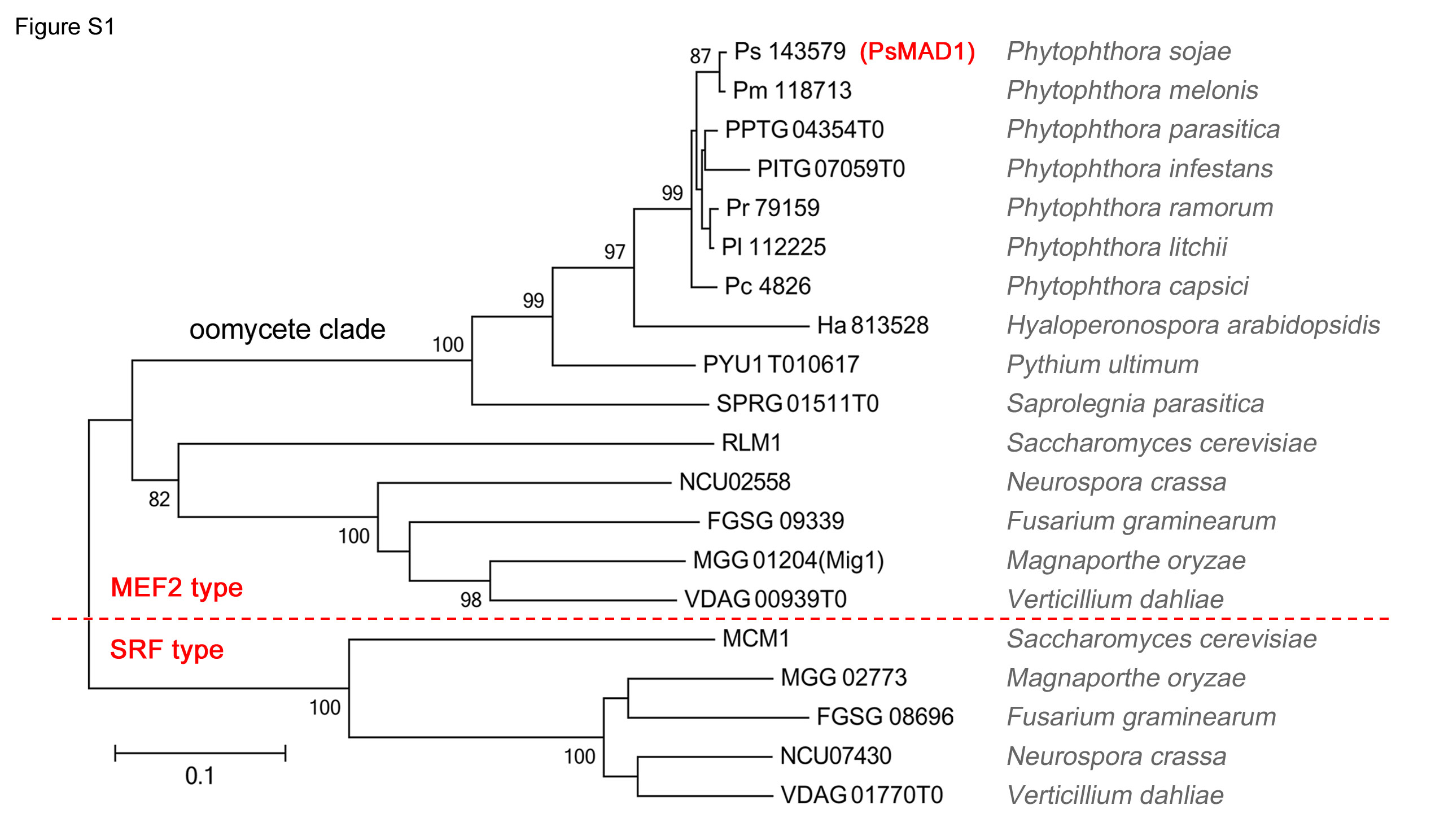

Supplement: FIGURE S1 — Phylogenetic analysis of putative MADS-box TF proteins. The neighbor-joining tree was constructed based on the amino acid sequences. Bootstrap values greater than 80 were displayed. [file Image_1.JPEG]

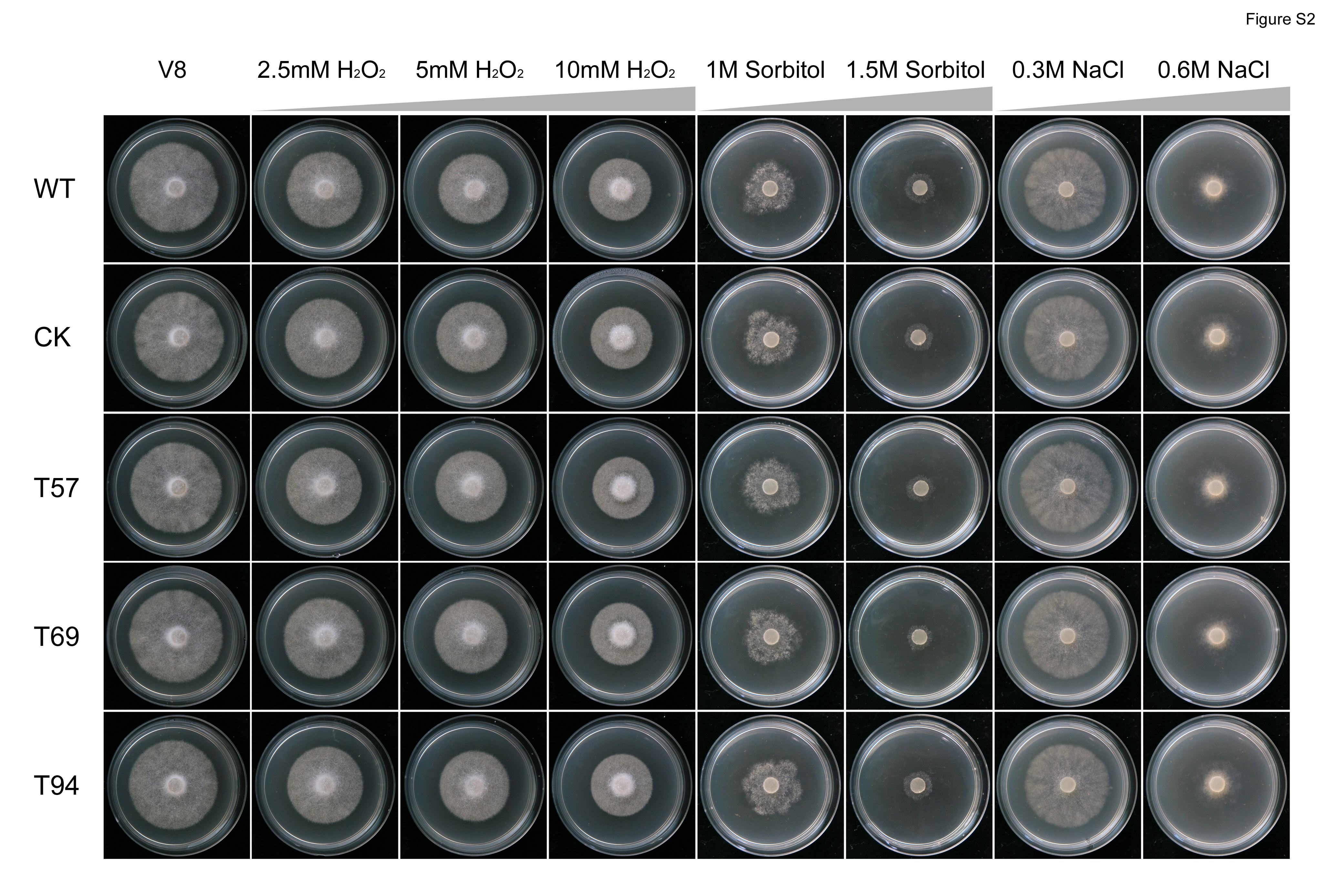

Supplement: FIGURE S2 — PsMAD1 mutants showed similar growth rate and sensitivity to H2O2, sorbitol, and NaCl as the wild type (WT). Mycelial growth of the WT, control (CK), and mutants on V8 medium plates without or supplemented with different concentration of H2O2, sorbitol, or NaCl. [file Image_2.JPEG]

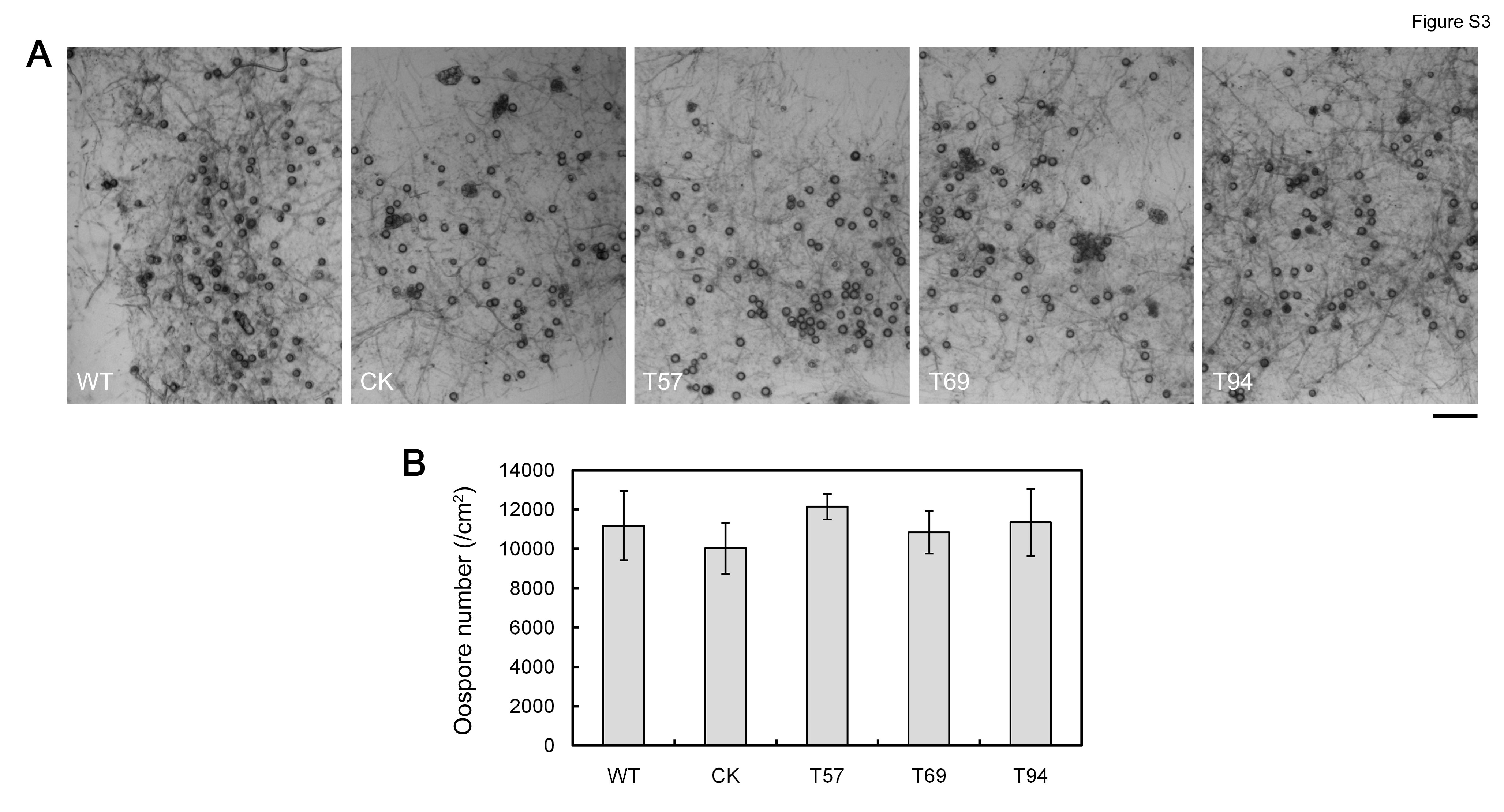

Supplement: FIGURE S3 — PsMAD1 mutants showed similar oospore production as the wild type (WT). (A) Oospore production of the WT, control (CK), and mutants. (B) Number of oospores per centimeter-squared zone around the inoculation site. All strains were grown for 10 days on lima bean agar medium to induce oospore formation. Scale bar = 200 μm. [file Image_3.JPEG]

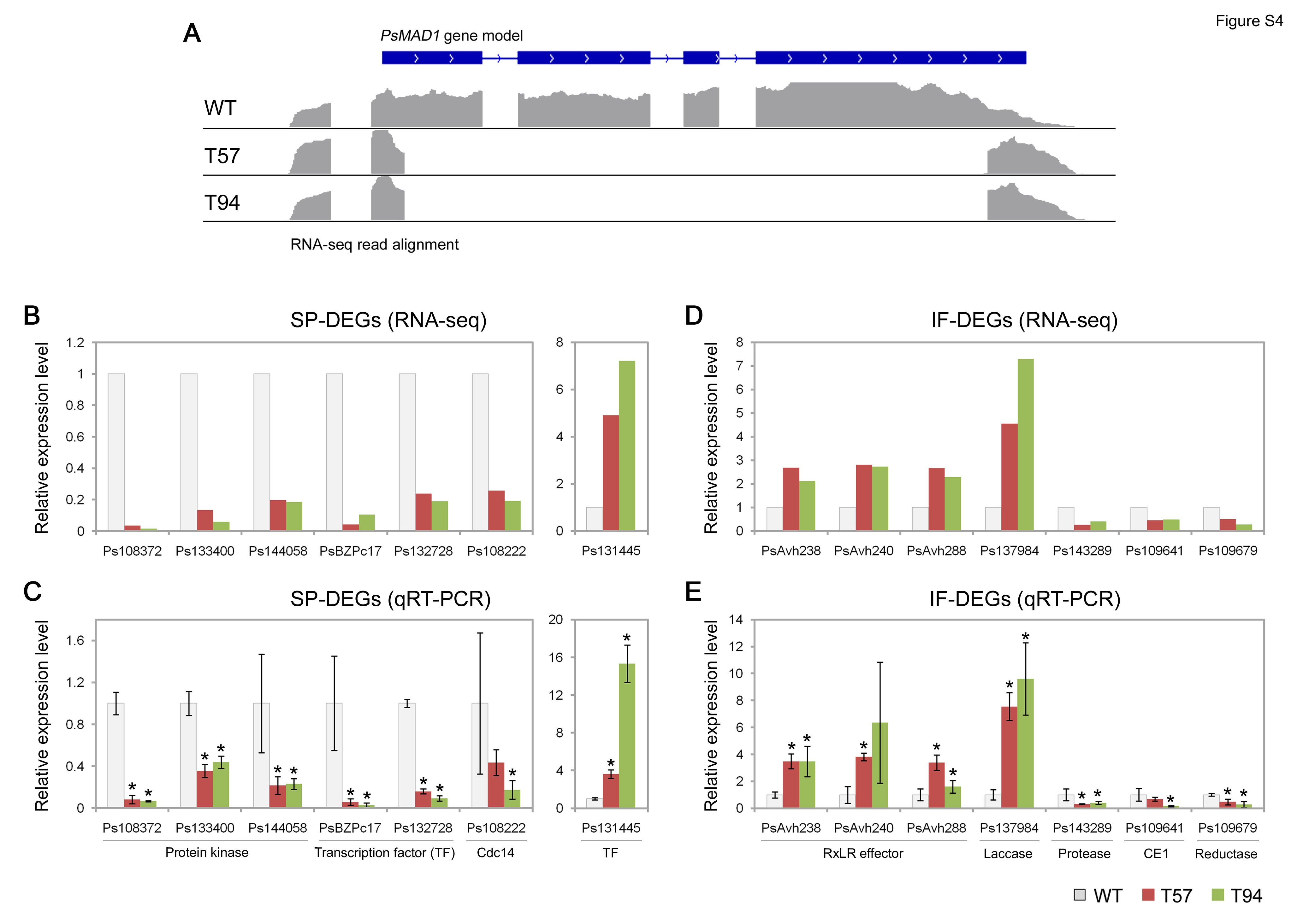

Supplement: FIGURE S4 — Transcriptional analysis and verification of indicated genes. (A) Transcript reads of PsMAD1 were truncated in mutant libraries. (B–E) Expression levels of selected genes were confirmed by qRT-PCR. Asterisks indicate significant differences (P < 0.01). [file Image_4.JPEG]

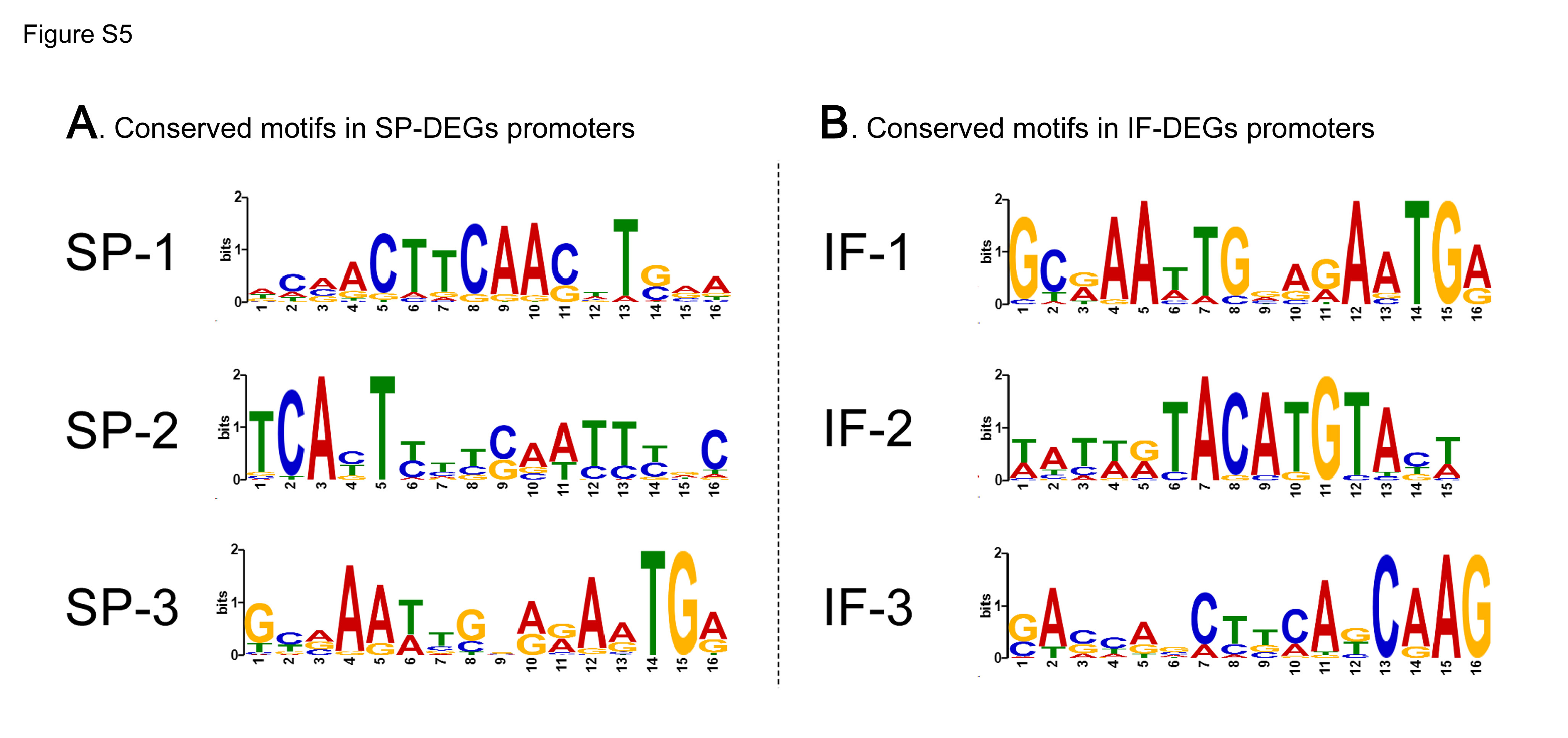

Supplement: FIGURE S5 — Identified conserved motifs in promoter sequences of PsMAD1-regulated genes. One kilobase promoter sequences of SP-DEGs (A) and IF-DEGs (B) were used to identify conserved motifs using MEME software, respectively. The top three motifs with most statistical significance were shown. [file Image_5.JPEG]
